# Diversity and Antimicrobial Potential of Cultivable Endophytic Actinobacteria Associated With the Medicinal Plant *Thymus roseus*

**DOI:** 10.3389/fmicb.2020.00191

**Published:** 2020-03-12

**Authors:** Zulpiya Musa, Jinbiao Ma, Dilfuza Egamberdieva, Osama Abdalla Abdelshafy Mohamad, Gulsumay Abaydulla, Yonghong Liu, Wen-Jun Li, Li Li

**Affiliations:** ^1^CAS Key Laboratory of Biogeography and Bioresource in Arid Land, Xinjiang Institute of Ecology and Geography, Ürümqi, China; ^2^Department of Medicine, College of Kashgar Vocational Technology, Kashgar, China; ^3^Faculty of Biology, National University of Uzbekistan, Tashkent, Uzbekistan; ^4^Department of Environmental Protection, Faculty of Environmental Agricultural Sciences, Arish University, Arish, Egypt; ^5^Xinjiang Laboratory of Resources Microbiology, College of Life Sciences and Technology, Xinjiang University, Ürümqi, China; ^6^State Key Laboratory of Biocontrol and Guangdong Provincial Key Laboratory of Plant Resources, School of Life Sciences, Sun Yat-sen University, Guangzhou, China

**Keywords:** endophytic actinobacteria, *Thymus roseus* Schipcz, diversity, antimicrobial activity, medicinal plant, environmental microbiology

## Abstract

We report for the first time the isolation of endophytic actinobacteria associated with wild populations of the Chinese medicinal herb *Thymus roseus* Schipcz obtained from the arid land in Ili and Tacheng of the Xinjiang Province, China. Strains were isolated by special pretreatment of plant tissues and identified based on their 16S rRNA gene sequences, and their antimicrobial activities *in vitro* were evaluated. A total of 126 endophytic actinobacteria belonging to two classes, eight orders, 14 families, and 24 genera were isolated from different organs at the Ili and Tacheng sites. In addition, the diversity of culturable endophytic actinobacteria genera was higher at Tacheng site (*n* = 71, 56.35%) than the Ili site (*n* = 55, 43.65%). A neighbor-joining tree of 126 isolated actinobacteria showing the phylogenetic relationships based on 16S rRNA gene sequences and the genus *Streptomyces* was the most dominant isolate. The number of endophytic actinobacteria genera obtained from root tissues (*n* = 54, 42.86%) was higher compared to stem (*n* = 35, 27.78%) and leaf tissue (*n* = 37, 29.36%). Among 126 endophytic actinobacteria, 54 strains were antagonistic against at least one or more indicator organisms *in vitro*. Notably, most strains of *Streptomyces* proved antagonistic activities. For example, strain T4SB028, namely *Streptomyces polyantibioticus*, showed the highest inhibition ratio reached 67.06, 64.20, and 70.55% against *Alternaria solani*, *Valsa malicola*, and *Valsa mali*, respectively. The results demonstrate that about 30.95%, 23.01% of the tested endophytic actinobacteria were capable of producing siderophores and chitinase, respectively. Additionally, the results of the amplification of biosynthetic genes polyketide synthetase (*PKS-I*) and non-ribosomal peptide synthetase (*NRPS*) indicated that at least one antibiotic biosynthetic gene was detected in 27 (50%) of the tested strains. Our result emphasizes that the endophytic actinobacteria communities are different based on the plant tissues and the geographical environment of the sampled area. Thus, we conclude that *T. roseus* Schipcz. provided a rich source of endophytic actinobacteria that exhibited a broad-spectrum antimicrobial agent.

## Introduction

Endophytes are microorganisms that inhabit healthy plant tissues at specific growth stages, or whole stages of their life cycle, and establish a symbiotic relationship with the host without causing any apparent disease symptoms (Petrini, 1991; Duan et al., 2013; Castro et al., 2014; Egamberdieva et al., 2017b, 2018; Li et al., 2018; Liu et al., 2019). Endophytic actinobacteria are considered to be a vital resource of microbial biodiversity (Matsumoto and Takahashi, 2017). A few reports have investigated a wide range of taxonomic status of endophytic actinobacteria associated with medicinal plants (Qin et al., 2009; Zhao et al., 2011; Matsumoto and Takahashi, 2017).

Endophytic actinobacteria exploit an unusual habitat may contribute to plant natural defenses by preventing herbivores and promoting the biocontrol of pathogens and pests due to their ability to produce the same or similar compounds with the host plants (Shimizu et al., 2009). Therefore, great attention has been paid by researchers toward their potential applications in pharmaceutical, agricultural, and food industries (Strobel and Daisy, 2003; Singh and Gaur, 2016). For instance, a total of 150 endophytic actinobacteria were isolated from three medicinal plant species (*Annonaceae squamosa*, *Camptotheca acuminata*, and *Taxus chinen*), and 72.4% of them showed inhibition against more than one indicator microorganism, including *Bacillus subtilis*, *Escherichia coli*, *Staphylococcus aureus*, *Candida albicans*, and *Aspergillus niger* (Wu et al., 2009). In addition, endophytic actinobacteria were antagonistic against *Pythium aphanidermatum* and significantly promoted plant growth and reduced the crown and root rot of cucumber that was caused by the *Pythium aphanidermatum in vitro* condition (El-Tarabily et al., 2009).

Medicinal plants in China have an ethnomedicinal history, and there are about 128,000 kinds of crude medicine resources (Cai et al., 2013). In other words, the vast majority of traditional Chinese medicine and natural chemical products had been derived from medicinal plants due to their effectiveness in various components for disease prevention and treatment. *Thymus roseus* Schipcz is one of the traditional Chinese medicinal herbs belonging to the *Labiatae* family, and it has a strong adaptive capability for a variety of particular habitats, such as arid lands, saline-alkali soils, and desertified soils (Ren et al., 2010). Furthermore, modern pharmacology has demonstrated that *Thymus* and thyme essential oils possess of anti-inflammatory, antibiosis, antiviral, antioxidant, anticancer, and antithrombus properties (Liang et al., 2014; Bobis et al., 2015; Jariæ et al., 2015; Hosseinzadeh et al., 2015). There is high value in medical treatment and chemical use of *T. roseus* S.; thus, the study of endophytic actinobacteria and their interaction with the host plants *Thymus* and its adaptive properties to arid lands is of great import within arid land ecology. This has the potential to significantly better our understanding of its diversity and antimicrobial activities as well as to further investigate its biotechnological applications potentials. However, the selection rationale of host plants, locations, and pretreatment methods for the isolation of endophytes is essential in biodiversity research (Strobel and Daisy, 2003; Zhang et al., 2016). Besides, endophytic resources of arid land are most likely to possess unique and diverse functions, such as degrading chitin, producing siderophore, encoding antimicrobial and antibiotic biosynthetic genes, because of their high ability to adapt the extremely arid environment (Qin et al., 2009; Cho et al., 2015; Egamberdieva et al., 2016, 2017c; Li et al., 2018). For instance, our research group reported that the vast majority of endophytic isolates from Chinese traditional medicinal plants *Ferula sinkiangensis*, *Glycyrrhiza uralensis*, and *Ferula songorica* in Xinjiang arid land were capable of producing both antifungal and plant growth-promoting traits (Liu et al., 2016, 2017; Li et al., 2018).

To the best of our knowledge, to date, there is no report on the diversity and antimicrobial potential of endophytic actinobacteria associated with *Thymus*. Thus, the objectives of our study were as follows: (1) to isolate and identify endophytic actinobacteria associated with Chinese traditional medicinal plant *Thymus roseus* Schipcz by a culture-dependent method*;* (2) to analyze the species richness and distribution pattern among the different plant tissues and locations; (3) to compare the effectiveness of plant tissue pretreatment on the isolation of endophytic actinobacteria; and (4) to determine their antimicrobial capability and to screen the isolates with the antibiotic biosynthetic genes in *in vitro* conditions.

## Materials and Methods

### Site Selection and Plant Sampling

**FIGURE 1:**
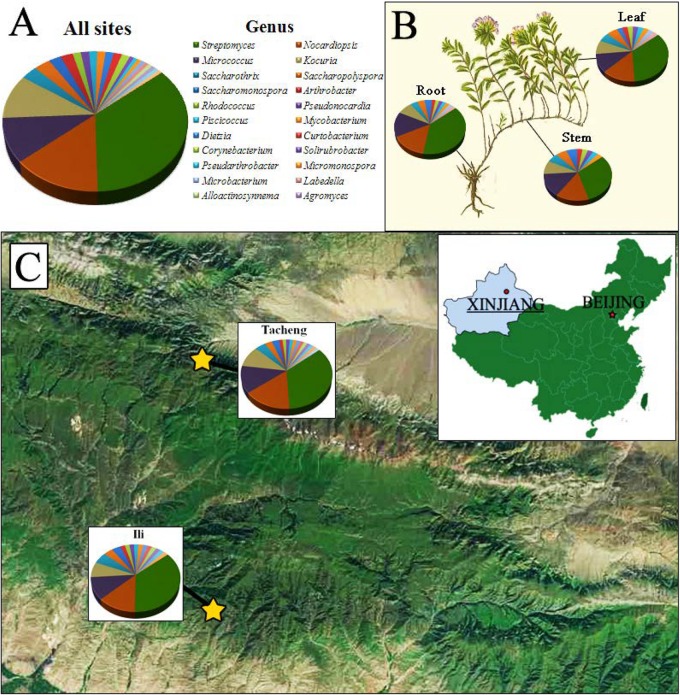
The distribution and identity of 126 culturable endophytic actinomycetes from *T. roseus* Schipcz. based on 16S rRNA gene sequences. **(A)** A summary of genera present at all sites. **(B)** Genus assignments for isolates from different tissues. **(C)** Genus assignments according to location and showing the high diversity of isolates from the most arid site, Tacheng.

Healthy medicinal plants of *T. roseus* Schipcz. were randomly collected from their natural arid habitats in the Xinjiang province of China. The study sites were located at Ili (43°21′56′′ N, 84°21′51′′ E; 2116.00 m.a.s.l., belonging to continental semi-dry climate.) and Tacheng (46°55′23′′ N; 83°16′59′′ E; 1035.80 m.a.s.l., belonging to temperate continental arid climate). On August 10th, 2016 (Figure 1), the healthy plants were brought to the laboratory inside a sterile paper bag and used to isolate for endophytic actinobacteria within 48 h of collection.

### Surface Sterilization, Pretreatment, and Isolation of Endophytic Actinobacteria

Each plant sample was washed with running tap water to remove surface clays and adhering epiphytes completely. The plant samples were separated into stem, root, and leaf segments by using sterile scissors. Subsequently, samples were washed by sonification for 30 min at 45 kHz to remove the dislodge soil and organic matter. The surface sterilization protocol included five steps: plant segments were rinsed in 0.1% Tween 20 for 1 min, sequentially immersed in 5% sodium hypochlorite (NaOCl) for 4 min leaf segments and 6 min for stem and root segments, washed for 10 min in 2.5% Na_2_S_2_O_3_, soaked in 70% ethanol for 4 min (leaves) and 6 min (stems and roots), and finally rinsed in 10% NaHCO_3_ for 10 min to disrupt the growth of fungi. After each treatment, samples were rinsed three times in sterile water. To confirm the success of the sterilization protocol, 0.2 m L^–1^ of the sterile distilled water from the final wash process of each tissue were placed onto International Streptomyces Project medium ISP_\_ 2 (Shirling and Gottlieb, 1966) and incubated at 28°C for 1 week. Finally, the samples were placed on sterilized filter paper and thoroughly dried in a sterile Petri dish under the laminar flow chamber for 24 h. After drying under sterile conditions, the sterilized samples were crushed in a sterilized commercial blender (Joyoung, JYL-C012) for 20 s (leaves) and 30 s for (stems and roots) (Qin et al., 2008; Li et al., 2018).

The plant tissues (roots, stems, and leaves) were introduced to three kinds of pretreatment in order to kill bacteria and isolate of endophytic actinobacteria following the method of Qin et al. (2011) with some modification: (1) surface sterilized plant tissues were directly taken to isolation; (2) freezing treatment involved plant tissues being frozen at –80 °C for 2 weeks; (3) high temperature treatment involved plant tissues drying at 100°C for 20 min.

For each pretreated sample (roots, stems, and leaves), about 1 g of tissue homogenate was weighed aseptically and macerated with a sterile mortar and pestle along with 9 mL sterile double distilled water. After precipitation for 10 min at room temperature, the supernatant was serially diluted (10^–2^ and 10^–3^), and a suspension of 0.2 ml was spread onto ten different selective isolation media (Li et al., 2018; Table 1). In order to inhibit the growth of bacteria and fungi, the isolation media were supplemented with antibiotics: nystatin 50 mg L^–1^, K_2_Cr_2_O_7_ 25 mg L^–1^ and nalidixic acid 25 mg L^–1^. All agar plates were performed three replicates for each dilution and then warped with parafilm and incubated at 28°C for 45 days before being monitored every 7 days for microbial growth. The colonies were observed and selected according to their characteristics and colony morphology. The purified actinobacteria isolates were stored on the ISP2 slope medium at 4°C and in 20% glycerol at −80°C (Li et al., 2018).

**TABLE 1:** Composition of the media used for the isolation of endophytic actinomycetes from medicinal plants.

Medium	Composition	References
M1	Tap water yeast extract (TWYE): yeast 0.25 g; K_2_HPO4 0.5 g; Agar 15 g; pH 7.2; distilled water 1000 mL	El-Shatoury et al. (2006)
M2	Cellulose-proline: cellulose 2.5 g; sodium pyruvate 2.0 g; proline 1.0 g; KNO_3_ 0.25 g; MgSO_4_⋅7H_2_O 0.2 g; K_2_HPO_4_ 0.2 g; CaCl_2_ 0.5 g; FeSO_4_⋅7H_2_O 0.01 g and agar 15 g; distilled water 1000 mL; pH (7.2–7.4)	Modified from Wang (2015)
M3	Sodium succinate-asparagine: sodium succinate 1.0; L-asparagine 1.0 g; KH_2_PO_4_ 0.9 g; K_2_HPO_4_ 0.6 g; MgSO_4_⋅7H_2_0 0.1 g; CaCl_2_ 0.2 g; KCl 0.3 g; FeSO_4_⋅7H_2_O 0.001 g; agar 15 g; distilled water 1000 mL; pH 7.2	Modified from Wang (2015)
M4	Xylan-asparagine: xylan 2.5 g; asparagine 1 g; K_2_HPO_4_ 0.5 g; KNO_3_ 0.25 g; MgSO_4_⋅7H_2_O 0.2 g; CaCl_2_ 0.5 g; FeSO_4_⋅7H_2_O 0.01 g and agar 15 g in 1000 mL distilled water; pH (7.2–7.4)	Qin et al. (2009)
M5	Sodium propionate-asparagine: sodium propionate 2 g; L-asparagine 1.0 g; NH_4_NO_3_ 0.1 g; KCl 0.1 g; MgSO_4_⋅7H_2_O 0.05 g; FeSO_4_⋅7H_2_O 0.05 g; plant extract (100 g plants was boiled in 1 L distilled water for 1 h and then filtered and concentrated to 100 mL) 1 mL; agar 15 g; distilled water 1000 mL; pH 7.2	Qin et al. (2009)
M6	Histidine-raffinose: histidine O.5 g; raffinose 2.5 g; K_2_HP0_4_.3H_2_0 1 g; MgS0_4_⋅7H_2_0 0.5 g; FeS0_4_⋅7H_2_0 0.01 g; CaCl_2_ 0.02 g; distilled water 1000 mL	Vickers et al. (1984)
M7	Humic-vitamin (HV): humic acid 1 g; Na_2_HP0_4_ 0.5 g; KCl 1.7 g; MgS0_4_⋅7H_2_0 0.05 g; CaCl_2_ 1 g; vitamins mixture 1 g; distilled water 1000 mL	Hayakawa (1990)
M8	Oatmeal agar (ISP3): oatmeal 20 g; saline standard solution 1 mL; distilled water 1000 mL	Shirling and Gottlieb (1966)
M9	Modified Gause No. l: starch 20 g; KNO_3_ 1 g; K_2_HP0_4_ 0.5 g; MgS0_4_⋅7H_2_0 0.5 g; NaCI 0.5 g; FeS0_4_⋅7H_2_0 0.01 g; distilled water 1000 mL	Shirling and Gottlieb (1966)
M10	Citrate agar: citric acid 0.12 g; NaNO_3_ 1.5 g; K_2_HPO_4_⋅3H_2_O 0.4 g; Mg SO_4_⋅7H_2_O 0.1 g; CaCl_2_⋅H_2_O 0.05 g; EDTA 0.02 g; Na_2_CO_3_ 0.2 g; agar 15 g; pH 7.2; distilled water 1000 mL	Zhang et al. (2013)

### Taxonomic Characterization and 16S rRNA Gene Sequencing

Genomic DNA was extracted using the microwave thermal shock method (Orsini and Romano-Spica, 2001) and the TIANamp Bacteria DNA Kit (TIANGEN BIOTECH). The microwave thermal shock method was used: fresh bacterial biomass (50 mg) was resuspended in 35 μl lysis solution (50 mM sucrose, 50 mM Tris–HCl pH 8.0, and 20 mM EDTA-Na) and 15 μl 20% SDS and then heated in a microwave oven for 90 s. The mixture was treated with 450 μl DNA extraction solution (100 mmol.L^–1^ Tris, 100 mmol.L^–1^ EDTA, 200 mmol.L^–1^ NaCl, 2% PVP, 3% CTAB, and pH 9.0) and then extracted twice with phenol/chloroform/isoamyl alcohol (25:24:1 v/v/v) (Sangon Biotech) followed by precipitation with 800 μl ethanol and 80 μl sodium acetate (3 mol L^–1^ and pH 4.8–5.2). The DNA pellet was washed with 70% ethanol, air-dried, resuspended in 50 μl deionized distilled water, and then stored at −20°C for further study. A 16S rRNA gene sequence was amplified using universal primers 27F (5′-AGAGTTTGATCCTGGCTCAG-3′) and 1492R (5′-GGTTACCTTGTTACGACTT-3′) (Wang et al., 2006). The PCR mixture (25 μl) contained 12.5 μl 2 × Taq PCR Master Mix procured from TIANGEN BIOTECH (Beijing, China), 2 μl of the DNA template, and 1 μl of each primer. We used an amplification protocol: denaturation took place at 95°C for 5 min, and it was followed by 35 cycles of denaturation at 94°C for 1 min, annealing at 56°C for 1.5 min, extension at 72°C for 2 min, and a final extension at 72°C for 10 min. PCR products were purified and sequenced by a commercial company, Sangon Biotech. The 16S rRNA gene sequences were compared with Ezbiocloud^1^ (Chun et al., 2007) and GenBank databases using BLAST software. Multiple sequence alignment was performed using the CLUSTAL X 1.83 program (Thompson et al., 1997), and a phylogenetic tree was generated by the neighbor-joining method using MEGA version 7.0 software (Kumar et al., 2016). The 16S rRNA gene sequences determined in this study were deposited in GenBank under accession numbers MN686679–MN686702(24), MN687832–MN687853(22), MN688237–MN688255(19), MN688672–MN688674(3), MN688677(1), MN688679–MN688680(2), MN686608–MN686629(22), MN686648–686678(31), and MNMN688648–688649(2). Reference sequences used are noted in the phylogenetic trees.

### Antagonistic Assays of Antifungal Activities *In vitro*

The antifungal activity of each endophyte actinobacteria was screened for antagonism against six pathogenic fungi (provided by Key Laboratory of Biogeography and Bioresource in Arid Land, Xinjiang Institute of Ecology and Geography, Chinese Academy of Sciences)—Tomato Fusarium wilt (*Fusarium oxysporum* f.sp.), Tomato leaf mildew [*Fulvia fulva*(Cooke)Cif.], Tomato Early Blight (*Alternaria solani*), Cotton fusarium wilt (*Fusarium oxysporum*), Apple Valsa Canker (*Valsa malicola*), and Apple Valsa Canker (*Valsa mali)*—by the plate confrontation method as described by Liu et al. (2017). Briefly, a fungal disk 5 mm in diameter containing 7-day-old mycelial growth was placed at the center of a 9 cm potato dextrose agar (PDA) plates.

The four actinobacteria disks cultured on ISP2 medium for 4–7 days at 28°C were placed onto the agar surface at four equidistant points, 2.5 cm from the plate periphery (Loqman et al., 2009). Plates with pathogenic fungi alone served as a control. All agar plates were wrapped with parafilm and incubated at 28 ± 2°C for 3–5 days and observed for the inhibition of the pathogen.

The antifungal activity of isolates was determined by calculating the growth inhibition ratio. The formula of growth inhibition ratio (%) was calculated by *I* =(*R_ 0_–R_*i*_*)/*R_*O*_* × 100%, where *R*_ 0_ is the radial of pathogen in control plates, and *R*_*i*_ is the radial of pathogen in test plates (Mohamad et al., 2018). Each experiment was conducted with three replicates.

### Antagonistic Activity Against Human Pathogenic Bacteria

A slightly modified method from Nie et al. (2012) for detecting antibacterial activity was used against the three common bacteria—*Staphylococcus aureus*, *Bacillus cereus*, and *Salmonella enteritidis*—provided by Key Laboratory of Biogeography and Bioresource in Arid Land, Xinjiang Institute of Ecology and Geography, Chinese Academy of Sciences. The bacteria were cultured for 1 night in Luria-Bertani LB medium at 37°C. The tested bacteria and endophytes isolates were each pre-cultured overnight, and 5 m L^–1^ of each culture was centrifuged at 5000 rpm for 10 min. The pellets were resuspended in sterile DDH_2_O and density adjusted to 10^8^ colony forming units CFU/mL by using Densicheck plus (Biomerieux, United States). A total of 100 μL of the typical bacteria cell were inoculated and evenly spread by sterile cotton swaps onto the surface of the LB medium, and then four 5 mm diameter pieces of sterile filter paper were placed on each corner of the agar plate. After this, 10 μL of each actinobacteria strain was added dropwise to the filter paper. All plates were wrapped with parafilm, incubated at 37 ± 2°C for 24 h, and observed for the inhibition of the common bacteria (Mohamad et al., 2018). Antibacterial activity was assessed by measuring the diameter of the clear zone of growth inhibition. An equivalent volume of sterile DDH_2_O instead of the endophytic actinobacteria was used as a negative control.

### Chitinase and Siderophore Production

Chitinase production was assessed by using a Colloidal chitin-supplemented medium, as described by Agrawal and Kotasthane (2012). The protocol for preparing the colloidal chitin was such that Chitin was hydrolyzed in concentrated hydrochloric acid by stirring at 4°C for 12 h, and this was followed by extraction of colloidal chitin in 200 mL^–1^ of ice-cold 99% ethanol, neutralization at room temperature for another 12 h, and then centrifugation at 5000 rpm for 10 min. The pellet was washed with distilled water by centrifugation at 5000 rpm for 5 min for several times until the smell of alcohol was removed entirely and the pH was natural. Chitinase medium composed of (L^–1^) 2.0 g of KH_2_PO_4_, 1.0 g of citric acid monohydrate, 0.3 g of MgSO_4_⋅7H_2_O, 3.0 g of (NH_4_)2SO_4_, 15 g of agar, 200 μL of Tween-80, and 4.5 g of colloidal chitin was supplemented with 0.15 g of bromocresol purple and then autoclaved at 121°C for 20 min. All agar plates were wrapped with parafilm and three replicates for each isolate, incubated at 25 ± 2°C for 3–7 days, and were observed for the formation of a colored zone around bacterial colony. Sterile chitin agar media was used as a control for bacterial growth.

Siderophore production was evaluated by incubating endophytic actinobacteria on Chrome azurol S (CAS) agar medium at 28°C for 7 days following the methods described by Li et al. (2018). An orange halo surrounding the actinobacteria colony indicates the tested strains have the siderophore-producing ability. The experiment was conducted with three replicates.

### Screening for Natural Product Biosynthetic Gene Clusters by PCR Method

Fifty-four strains showed antimicrobial activity against one or more indicator organisms and were used for screening for natural product biosynthetic gene clusters by PCR. Three sets of degenerate primers targeting biosynthetic genes were used for PCR amplification: KSF (5′-GTSCCSGTSSCRTGSSHYTCSA-3′) and KSR (5′-CGCTCCATGGAYCCSCARCA-3′), which target *PKS-I* KS and methyl malonyl transferase domains (Kun et al., 2010); KSαF (5′-TSGCSTGCTTGGAYGCSATC-3′) and KSαR (5′-TGGAANCCGCCGAABCCGCT-3′), which target *PKS-II* KSα genes (Metsä-Ketelä et al., 1999); and A3F (5′-GCSTACSYSATSTACACSTCSGG-3′) and A7R (5′-SASGTCVCCSGTSCGGTAS-3′), which target non-ribosomal peptide synthetase (*NRPS*) genes. A PCR amplification protocol as described by Qin et al. (2009) was followed.

### Data Analysis

To study the level of significance, statistical analysis was made by one-way ANOVA using SPSS ver. 16.0 software. Mean comparison carried out by using Duncan’s multiple range tests at *P* < 0.05. Data are presented as means ± SD.

## Results

### Effectiveness of Surface Sterilization

No microbial growth was detected on the isolation media after 7 days of incubation at 28°C when the distilled water used in the final rinse of surface-sterilization was plated. This result indicated that the five-step surface sterilization protocol was successful, and all isolates were considered to be true endophytes.

### Pretreatment, Isolation Medium, and Effectiveness of Isolation

**FIGURE 2:**
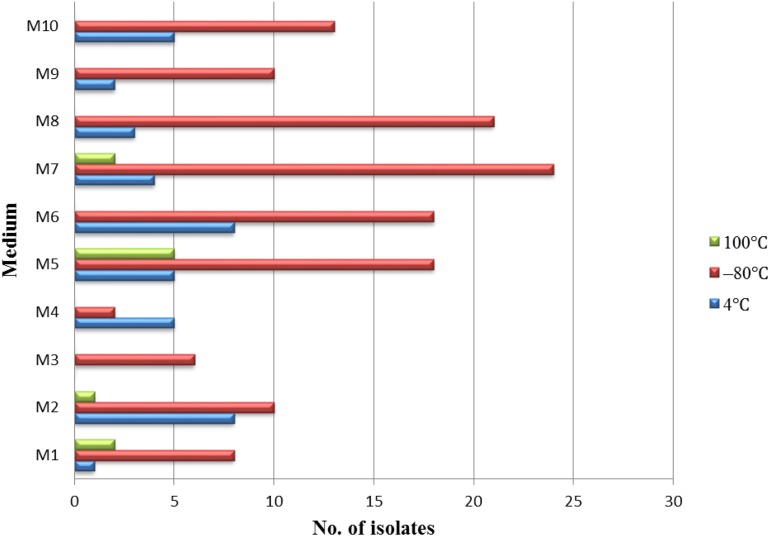
The isolation effectiveness of endophytes under the different pretreatments and medium.

Different pretreatment and media have been used for the isolation of endophytic actinobacteria. A total of 181 endophytic actinomycetes were isolated from three types of pretreatment of medicinal plant *T. roseus* S tissues. The 128, 41, and 10 strains were isolated from the plant tissues treated with freezing (–80°C), and this was followed by average temperature (4°C) and high-temperature treatment (100°C). Freezing treatment showed the highest number of isolations with comparing to the other two treatments (Figure 2). The effect of different media on the isolation of endophytic actinobacteria used in this study showed dramatic results; the majority of isolations were from humic acid-vitamin (HV) medium M7, followed by sodium propionate agar medium M5, raffinose-histidine agar medium M6, and oatmeal agar medium M8 (Figure 2).

### Diversity and Tissues Specificity of Endophytic Actinobacteria

**FIGURE 3:**
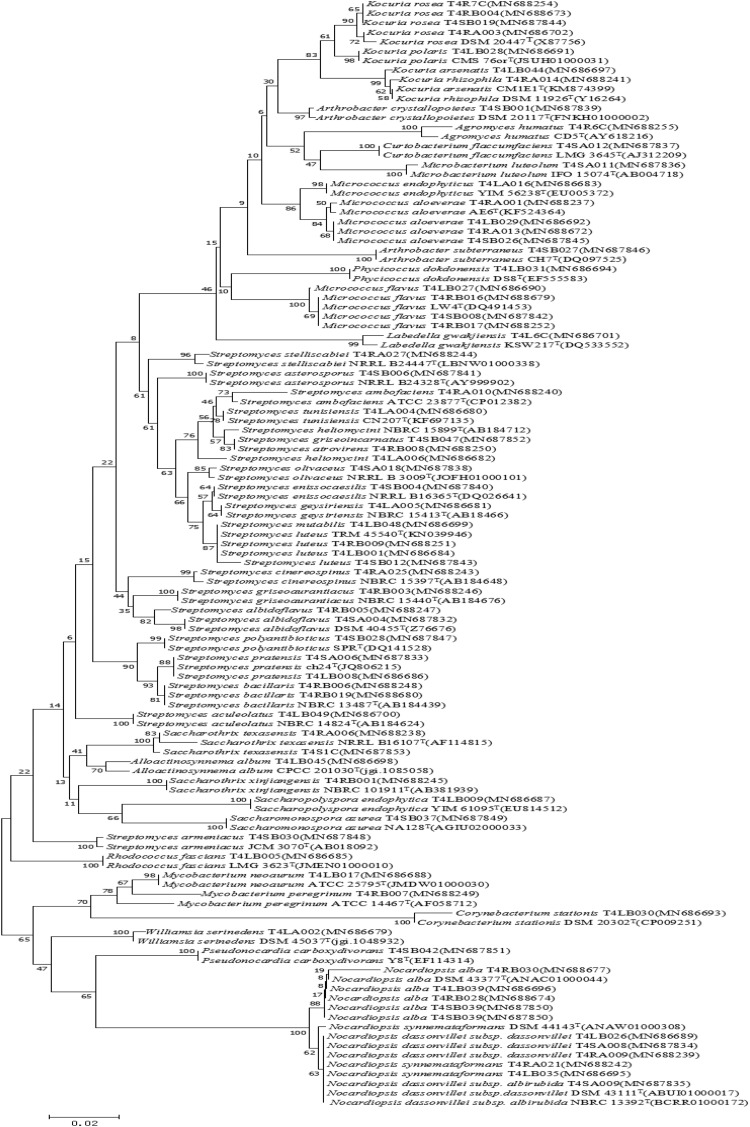
Neighbor-joining tree based on 16S rRNA gene sequences showing relationships between the endophytic actinomycetes (isolated from *T. roseus* Schipcz. in Tacheng) and the nearest type strains. The bar represents 2% sequence divergence.

**FIGURE 4:**
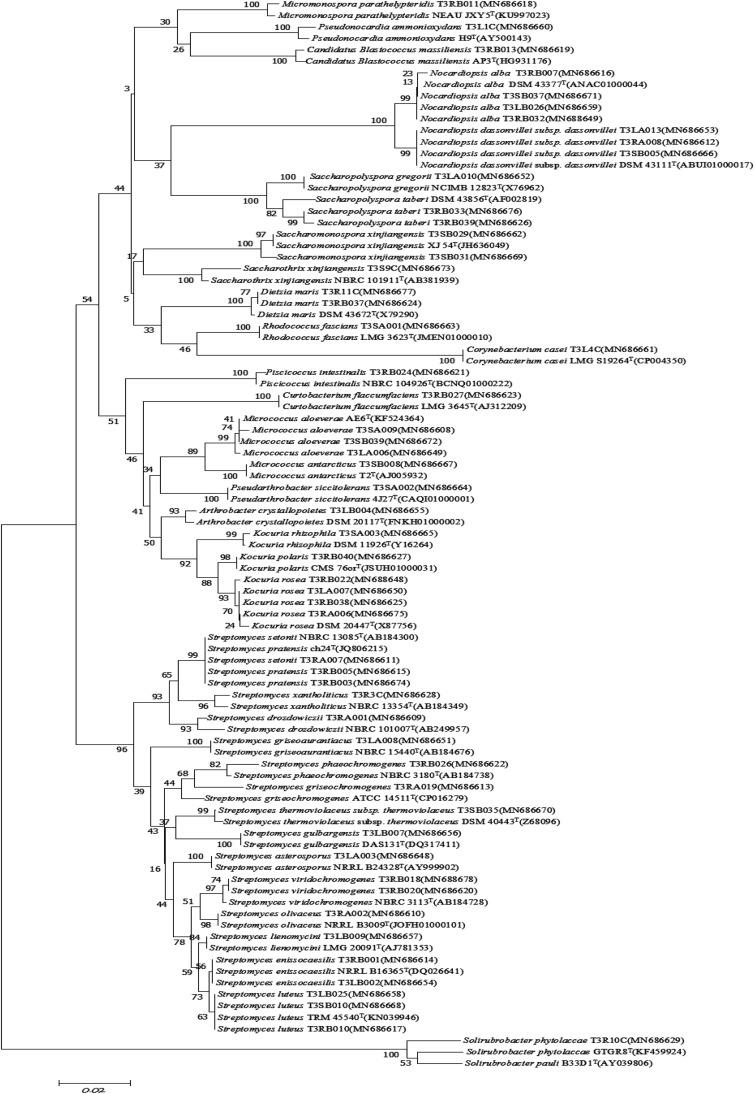
Neighbor-joining tree based on 16S rRNA gene sequences showing relationships between the endophytic actinomycetes (isolated from *T. roseus* Schipcz. in Ili) and the nearest type strains. The bar represents 2% sequence divergence.

A total of 126 endophytic actinobacteria isolates were obtained from the *T. roseus* S based on 16S rRNA gene sequences, and 55 and 71 isolates were from Ili and Tacheng sites, respectively. The diversity of culturable endophytic actinobacteria were the highest at the Tacheng site and included one class, five orders, nine families, 19 genera, and 49 species (Figure 3). While isolates from the Ili site included two classes, eight orders, 12 families, 18 genera, and 37 species (Figure 4). The endophytic actinobacteria community clearly differed in composition with regard to geography.

**FIGURE 5:**
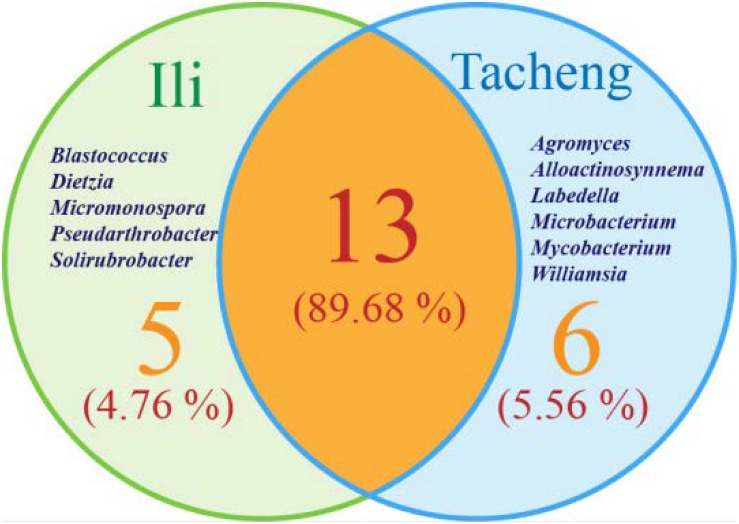
Venn diagram of endophytic actinomycetes in genera level from the *T. roseus* Schipcz. collected in two different geographical environments.

The isolates from both sites grouped into 24 genera with a predominance of *Streptomyces*, followed by *Nocardiopsis*, *Micrococcus*, *Kocuria*, and others. Among these isolates, the number of these genera was four times as predominant from Tacheng than at the Ili site (Figure 1). Six rare genera representative 5.56 % of all genera obtained—*Agromyces*, *Alloactinosynnema*, *Labedella*, *Microbacterium*, *Mycobacterium*, and *Williamsia*—were isolated only from Tacheng, while the five rare genera representative 4.76% of all genera obtained—*Blastococcus*, *Dietzia*, *Micromonospora*, *Pseudarthrobacter*, and *Solirubrobacter*—were isolated only from Ili site. These results indicated that there are some differences in the composition of endophytes from the same plant species in different geographical sites (Figure 5).

In this research, the endophytic actinomycete isolates displayed considerable diversity. The diversity parameters of each sample were different, and the values of the Shannon H indices (3.7205) and Margalf (11.26) of the species from Tacheng were higher than in the Ili site, while the Evenness (0.8460) was lower than the Ili site. All the indices clearly demonstrated that the diversity of endophytic actinomycete communities from Tacheng was more abundant than in Ili (Table 2).

**TABLE 2:** Diversity of endophytic actinomycetes isolated from *T. roseus* S. of Ili and Tacheng.

	Taxa_S	Individuals	Shannon_H	Evenness	Margalef
Ili	37	55	3.475	0.8726	8.98
Tacheng	49	71	3.725	0.846	11.26

Figure 1 exhibited that the genera to which strains from each sampled area and tissues belonged as well as the quantity of endophytes from each genus. The abundance of endophytic actinobacteria isolates in root tissues (*n* = 54, 42.86%) was significantly higher than in stems (*n* = 35, 27.78%) and leaves (*n* = 37, 29.37%), especially the isolates belonged to genera *Streptomyces*, *Nocardiopsis*, and *Kocuria*. However, the distribution of the genus *Micrococcus* was relatively similar in three kinds of tissues.

### Antimicrobial Activity of the Isolates

The 126 isolated endophytic actinobacteria from medicinal plant *T. roseus* S were tested, using plate confrontation assays, for their antibacterial and antifungal properties in *in vitro* conditions. Among these tested isolates, 54 strains were showed antimicrobial activity against one or more indicator pathogens (Table 3). The endophytes actinobacteria varied in their ability to inhibit the growth of the fungi, with the percentage of inhibition.

**TABLE 3:** Antimicrobial activity of 54 endophytic actinomycetes on indicated fungal and bacterial strains.

Strain no.	Species	Host	*F. oxysporum* f.sp.	*F. fulva*	*A. solani*	*F. oxysporum*	*V. malicola*	*V. mali*	*S. aureus*	*B. cereus*	*S. enteritidis*
			Inhibition rate/% (inhibition activity)	Inhibition rate/% (inhibition activity)	Inhibition rate/% (inhibition activity)	Inhibition rate/% (inhibition activity)	Inhibition rate/% (inhibition activity)	Inhibition rate/% (inhibition activity)	Inhibition activity	Inhibition activity	Inhibition activity
T3L1C	*P. ammonioxydans*	*T.roseus* Schipcz. from Ili	–	–	–	–	–	–	–	+	–
T3LA003	*S. asterosporus*		28.62% (+)	25.05% (+)	39.59% (++)	–	–	18.76% (+)	–	–	–
T3LB002	*S. enissocaesilis*		–	–	23.80% (+)	–	–	24.13% (+)	–	–	+
T3LB009	*S. lienomycini*		30.76% (+)	42.57% (+ +)	43.99% (+ +)	24.79% (+)	39.17% (+ +)	41.64% (+ +)	–	–	–
T3LB025	*S. luteus*		26.58% (+)	44.90% (+ +)	51.03% (+ + +)	25.76% (+)	–	58.14% (+ + +)	–	–	–
T3RA001	*S. drozdowiczii*		19.39% (+)	–	30.05% (+)	20.76% (+)	–	–	–	–	–
T3RA002	*S. olivaceus*		–	–	31.48% (+)	–	43.15% (+ +)	–	–	–	+ +
T3RA006	*K. rosea*		–	–	–	–	38.75% (+ +)	–	–	–	–
T3RA007	*S. setonii*		–	–	–	–	42.56% (+ +)	26.98% (+)	–	–	–
T3RA008	*N. dassonvillei* subsp. *dassonvillei*		–	–	–	–	62.00% (+ + +)	–	–	–	–
T3RB001	*S. enissocaesilis*		–	32.59% (+)	27.79% (+)	–	40.90% (+ +)	39.54% (+ +)	–	–	–
T3RB003	*S. pratensis*		–	30.48% (+)	–	–	48.25% (+ +)	25.63% (+)	–	–	–
T3RB005	*S. pratensis*		20.06% (+)	20.45% (+)	34.78% (+ +)	–	46.01% (+ +)	29.34% (+)	–	–	–
T3RB007	*N. alba*		–	12.15% (+)	–	–	–	–	+	+	+
T3RB010	*S. luteus*		27.18% (+)	44.48% (+ +)	50.37% (+ + +)	27.10% (+)	58.76% (+ + +)	61.30% (+ + +)	–	–	–
T3RB018	*S. viridochromogenes*		–	28.41% (+)	28.81% (+)	21.88% (+)	–	–	–	–	–
T3RB020	*S. viridochromogenes*		–	25.06% (+)	30.10% (+)	22.45% (+)	–	–	–	–	–
T3RB027	*C. flaccumfaciens*		–	–	–	–	35.71% (+ +)	33.20% (+)	–	–	–
T3RB033	*Sac. taberi*		16.20% (+)	27.30% (+)	28.50% (+)	–	32.71% (+)	–	+	++	++
T3RB038	*K. rosea*		–	–	–	–	–	30.11% (+)	–	–	–
T3RB039	*Sac. taberi*		–	–	–	–	24.66% (+)	–	–	–	–
T3RB040	*K. polaris*		–	–	–	–	–	28.23% (+)	–	–	–
T3SA002	*P. siccitolerans*		27.29% (+)	–	30.90% (+)	21.78% (+)	–	–	–	–	–
T3SB005	*N. dassonvillei* subsp. *dassonvillei*		–	–	–	–	–	–	+	++	+++
T3SB010	*S. luteus*		–	29.75% (+)	17.39% (+)	–	40.83% (+ +)	31.18% (+)	–	–	–
T3SB029	*Sac. xinjiangensis*		26.50% (+)	–	21.01% (+)	–	–	32.28% (+)	–	–	–
T3SB035	*S. thermoviolaceus* subsp. *thermoviolaceus*		–	–	–	–	–	–	+	+	+ + +
T3SB037	*N. alba*		–	–	–	–	–	–	+	+	+
T4LA005	*S. geysiriensis*	*T. roseus* Schipcz. from Tcheng	–	39.90% (+ +)	–	–	–	38.33% (+ +)	–	–	–
T4LA006	*S. heliomycini*		27.19% (+)	–	–	20.12% (+)	–	–	–	–	–
T4LB001	*S. luteus*		30.88% (+)	44.65% (+ +)	52.03% (+ + +)	26.76% (+)	–	56.15% (+ + +)	–	–	–
T4LB008	*S. pratensis*		–	25.14% (+)	–	28.90% (+)	–	–	–	–	–
T4LB009	*Sac. endophytica*		–	–	42.81% (+ +)	–	40.99% (+ +)	19.90% (+)	–	–	–
T4LB039	*N. alba*		–	–	29.43% (+)	–	–	–	–	–	–
T4LB045	*A. album*		–	17.05% (+)	29.81% (+)	–	40.95% (+ +)	–	+	–	+
T4LB048	*S. mutabilis*		26.09% (+)	45.17% (+ +)	39.06% (+ +)	24.06% (+)	59.81% (+ + +)	44.95% (+ +)	–	–	–
T4LB049	*S. aculeolatus*		–	–	–	–	–	–	+ +	++	++
T4RA014	*K. rhizophila*		–	–	–	21.10% (+)	–	–	–	–	–
T4RA021	*N. synnemataformans*		–	–	–	–	45.52% (+ +)	–	–	–	–
T4RA027	*S. stelliscabiei*		26.71% (+)	–	31.31% (+)	25.09% (+)	48.88% (+ +)	48.59% (+ +)	–	–	–
T4RB005	*S. albidoflavus*		35.32% (+ +)	55.29% (+ + +)	63.06% (+ + +)	27.62% (+)	61.42% (+ + +)	51.75% (+ + +)	–	–	+
T4RB006	*S. bacillaris*		–	–	–	–	45.28% (+ +)	36.45% (+ +)	–	–	–
T4RB008	*S. atrovirens*		–	–	–	–	52.81% (+ + +)	–	–	–	–
T4RB009	*S. luteus*		–	–	–	–	42.50% (+ +)	20.94% (+)	–	–	–
T4RB028	*N. alba*		–	–	–	–	–	–	+	–	+
T4SA004	*S. albidoflavus*		21.16% (+)	38.38% (+ +)	53.92% (+ + +)	31.91% (+)	56.56% (+ + +)	55.59% (+ + +)	–	–	–
T4SA006	*S. pratensis*		34.31% (+ +)	11.91% (+)	31.17% (+)	35.37% (+ +)	–	33.14% (+)	–	–	–
T4SA008	*N. dassonvillei* subsp. *dassonvillei*		19.15% (+)	–	–	–	–	–	–	–	–
T4SA018	*S. olivaceus*		21.17% (+)	31.22% (+)	28.55% (+)	–	42.27% (+ +)	29.27% (+)	–	–	–
T4SB004	*S. enissocaesilis*		–	–	–	–	62.77% (+ + +)	33.24% (+)	–	–	+
T4SB012	*S. luteus*		–	–	–	22.61% (+)	52.22% (+ + +)	34.32% (+ +)	–	–	–
T4SB028	*S. polyantibioticus*		34.89% (+ +)	48.96% (+ +)	67.06% (+ + +)	29.38% (+)	64.20% (+ + +)	70.55% (+ + +)	–	+	+
T4SB030	*S. armeniacus*		38.08% (++)	53.88% (+++)	53.59% (+++)	38.56% (++)	64.20% (+++)	58.98% (+++)	++	++	++
T4SB037	*Sac. azurea*		–	–	52.99% (+ + +)	–	17.17% (+)	13.84% (+)	–	–	–

*Antifungal activity: inhibition zone width < 3 mm: “–”means negative (3.00–10.00) mm; “ + ”means weak (10.00–15.00) mm; “ + + ”means medium ≥15 mm; “ + + + ”means strong; antibacterial activity: inhibition zone width < 0 mm; “–”means negative (0.00–5.00) mm; “ + ”means weak (5.00–10.00) mm; “ + + ”means medium (10.00–15.00) mm: “ + + + ” means strong.*

**FIGURE 6:**
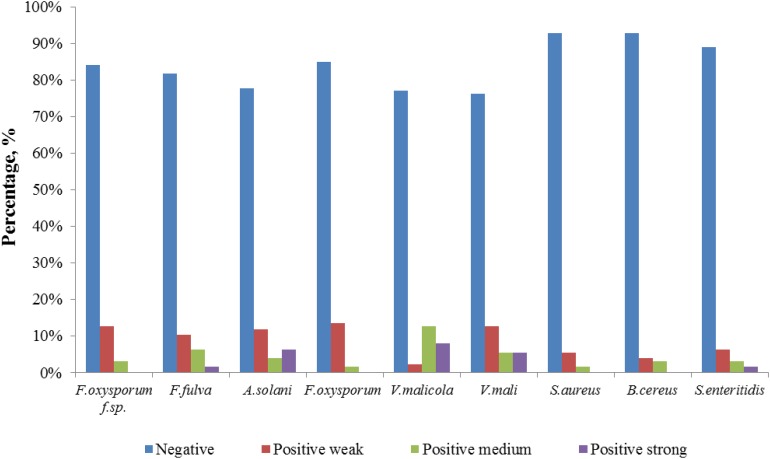
Distribution of positive strains tested for antimicrobial potential.

Comparatively potent activities against *Fulvia fulva* (Cooke) Cif., *Alternaria solani*, *Valsa malicola*, *Valsa mali*, and *Salmonella enteritidis* were identified in 1.59, 6.35, 7.94, 5.56, and 1.59% of the isolates, respectively. In contrast, all tested strains exhibited no potent inhibition against *Fusarium oxysporum* f.sp. and *Fusarium oxysporum*, and they merely showed moderate and weak activities. Of the 126 isolates, few strains showed high activities against human pathogens (Figure 6). Strain T3SB005 revealed completely antagonistic activity against all the three human pathogens of which T3SB005 showed a sizeable clear zone of inhibition to *Salmonella enteritidis* (+++) (Table 3).

Among these positive isolates, 14 strains, including T4RB005, T3RB010, T4SA004, T4SB028, T4LB048, T4LB001, T3SB005, T3RB033, T4SA006, T3RB005, T4SB004, T3RB001, and T4LB045, showed a high and broad antimicrobial spectrum. Strain T4SB030 namely *Streptomyces armeniacus* had an inhibitory effect on the growth of all nine tested organisms. Moreover, strain T4SB028, namely *Streptomyces polyantibioticus*, showed inhibition against all the pathogens except for *Staphylococcus aureus*, and the inhibition ratio of T4SB028 reached 67.06, 64.20, and 70.55% against *Alternaria solani*, *Valsa malicola*, and *Valsa mali*, respectively. The statistical analysis revealed that the highest inhibition activity against *Valsa mali* was shown by T4SB028 (*P* < 0.05) (Tables 3,4).

**TABLE 4:** The determination of antifungal spectrum of 13 potent antagonistic strains.

Strain no.	Species	Similarity	*F. oxysporum* f.sp.	*F. fulva*	*A. solani*	*F. oxysporum*	*V. malicola*	*V. mali*	*S. aureus*	*B. cereus*	*S. enteritidis*
T4SB030	*S. armeniacus*	99.61%	11.57 ± 0.40a	16.2 ± 0.33a	15.96 ± 0.51b	11.48 ± 0.34b	19.09 ± 0.03a	17.47 ± 0.01b	8.31 ± 0.61a	9.75 ± 1.70a	9.17 ± 0.32b
T4RB005	*S. albidoflavus*	99.22%	10.73 ± 0.52a	16.59 ± 0.51a	18.78 ± 0.50a	8.19 ± 0.08ed	18.43 ± 0.58ab	15.33 ± 0.38d	0.00 ± 0.00	0.00 ± 0.00	2.13 ± 0.20d
T3RB010	*S. luteus*	100.00%	8.41 ± 0.26cd	13.33 ± 0.12c	15.00 ± 0.57b	8.12 ± 1.20ed	17.48 ± 0.04bc	18.23 ± 0.49b	0.00 ± 0.00	0.00 ± 0.00	0.00 ± 0.00
T4SA004	*S. albidoflavus*	100.00%	6.43 ± 0.31e	11.52 ± 0.23d	16.06 ± 1.09b	9.48 ± 1.08cd	16.82 ± 0.62c	16.44 ± 0.93c	0.00 ± 0.00	0.00 ± 0.00	0.00 ± 0.00
T4SB028	*S. polyantibioticus*	99.80%	10.6 ± 0.40a	14.69 ± 0.09b	19.97 ± 1.33a	8.72 ± 0.27cde	19.09 ± 0.09a	20.94 ± 0.06a	0.00 ± 0.00	2.32 ± 0.10c	1.69 ± 0.44d
T4LB048	*S. mutabilis*	100.00%	7.93 ± 0.44d	13.56 ± 1.06c	11.63 ± 1.01c	7.12 ± 0.49e	17.77 ± 1.13abc	13.20 ± 0.78e	0.00 ± 0.00	0.00 ± 0.00	0.00 ± 0.00
T4LB001	*S. luteus*	100.00%	9.38 ± 0.51b	13.4 ± 0.48c	15.49 ± 0.39b	7.93 ± 1.39ed	0.00 ± 0.00	16.64 ± 0.51c	0.00 ± 0.00	0.00 ± 0.00	0.00 ± 0.00
T3SB005	*N. dassonvillei* subsp. *dassonvillei*	100.00%	0.00 ± 0.00	0.00 ± 0.00	0.00 ± 0.00	0.00 ± 0.00	0.00 ± 0.00	0.00 ± 0.00	3.68 ± 0.97b	6.97 ± 1.32b	12.95 ± 0.19a
T3RB033	*Sac. taberi*	98.48%	5.09 ± 0.26f	8.16 ± 0.48e	8.49 ± 0.50e	0.00 ± 0.00	9.73 ± 0.01f	0.00 ± 0.00	2.41 ± 0.25c	5.05 ± 1.19b	6.52 ± 0.69c
T4SA006	*S. pratensis*	100.00%	10.43 ± 1.68ab	3.57 ± 0.12h	9.28 ± 0.28ed	10.45 ± 0.24bc	0.00 ± 0.00	9.77 ± 0.29f	0.00 ± 0.00	0.00 ± 0.00	0.00 ± 0.00
T3RB005	*S. pratensis*	100.00%	6.26 ± 0.44e	6.11 ± 0.19f	10.36 ± 1.03d	0.00 ± 0.00	13.68 ± 1.35d	8.73 ± 0.15g	0.00 ± 0.00	0.00 ± 0.00	0.00 ± 0.00
T4SB004	*S. enissocaesilis*	100.00%	0.00 ± 0.00	0.00 ± 0.00	0.00 ± 0.00	0.00 ± 0.00	18.67 ± 0.01ab	9.8 ± 0.05f	0.00 ± 0.00	0.00 ± 0.00	2.18 ± 0.27d
T3RB001	*S. enissocaesilis*	100.00%	6.05 ± 0.64ef	0.00 ± 0.00	3.38 ± 0.23f	15.38 ± 1.82a	0.00 ± 0.00	0.00 ± 0.00	0.00 ± 0.00	0.00 ± 0.00	0.00 ± 0.00
T4LB045	*A. album*	97.51%	0.00 ± 0.00	5.12 ± 0.07g	8.88 ± 0.03e	0.00 ± 0.00	12.18 ± 1.19e	0.00 ± 0.00	2.06 ± 0.14c	0.00 ± 0.00	1.81 ± 0.17d

*Data are given as means; the determination of lowercase letters in the same column shows a significant difference at the 0.05 level (*P* < 0.05).*

**FIGURE 7:**
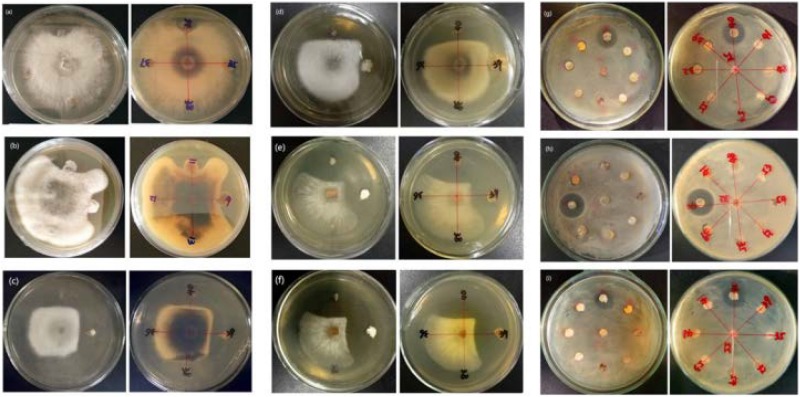
Representation of antimicrobial activities by the endophytic actinomycetes isolated from *T. roseus* Schipcz. **(a)** biocontrol activity against *F. oxysporum* f.sp; **(b)** biocontrol activity against *F. fulva* (Cooke) Cif.; **(c)** biocontrol activity against *A. solani*; **(d)** biocontrol activity against *F. oxysporum*; **(e)** biocontrol activity against *V. malicola*; **(f)** biocontrol activity against *V. mali*; **(g)** biocontrol activity against *S. enteritidis*; **(h)** biocontrol activity against *B. cereus*; **(i)** biocontrol activity against *S. aureus*; and 39 and 40 represent T4SB028 (*S. armeniacus*) and T4SB030 (*S. polyantibioticus*), respectively.

Of the 14 antagonistic strains, T3RB005 and T4LB045 displayed antagonisms against at least five indicator organisms. T3RB005 exhibited 98.48% 16S rRNA gene sequence similarity with the *Saccharopolyspora taberi*, while strain TLB045 displayed 97.51 % 16S rRNA gene sequence similarity with *Alloactinosynnema album* (Table 4). The active strains exhibited some selectivity in terms of some pathogens. Strain T4SB004 inhibited the growth of three pathogens, including *Valsa malicola*, *Valsa mali*, and *Salmonella enteritidis*; in a similar way, strain T3RB001 inhibited the growth of *Fusarium oxysporum* f.sp., *Fusarium oxysporum*, and *Alternaria solani*. Moreover, these two strains exhibited 100 % similarity with *Streptomyces enissocaesilis*; however, the two strains isolated from *T. roseus* Schipcz. in Ili and Tacheng, respectively (Tables 3, 4). This result revealed that the strains with the same taxonomy—but that are isolated from different geographic sites—showed different antagonistic activity, which can be further exemplified by the statistical analysis between strain T4SA006 and T3RB005 as well as strain T3RB010 and T4LB001 (Table 4). A representative picture illustrating the antagonistic activities is shown in Figure 7.

### Screening of Chitinase- and Siderophore-Producing Strains

**FIGURE 8:**
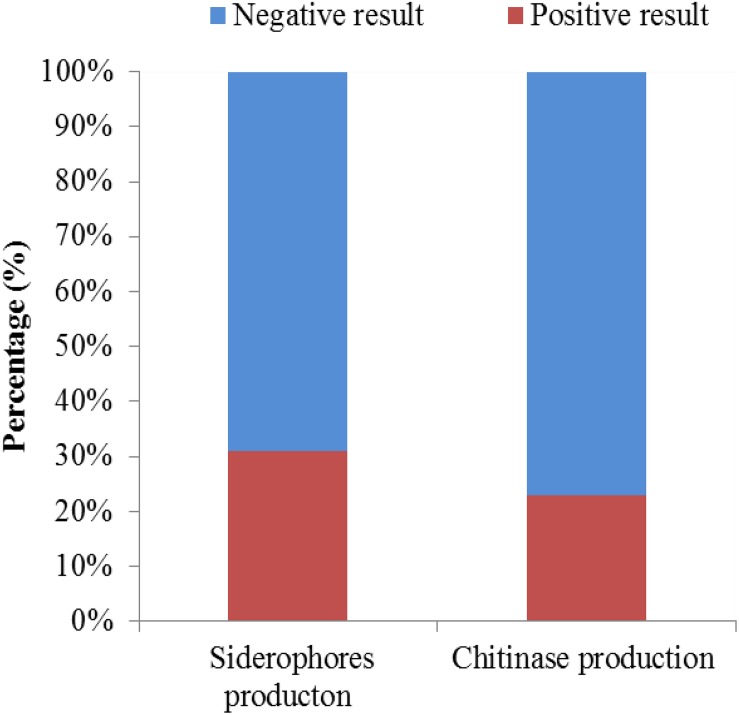
The chitinase- and siderophore-producing activities of isolates.

A total of 126 endophytic actinomycete isolates were screened for their siderophores and chitinase production abilities (Figure 8). Around 30.95 and 23.01% of the tested strains produced siderophores and chitinase, respectively.

### Screening of Isolates With Antibiotics Biosynthetic Genes

In this study, 27 out of the 54 strains possessed at least one of the test genes. Among the 54 antagonistic strains examined, 4 (7.4 %), 21 (38.9 %), and 15 (27.8 %) were identified as positive of *PKS I*, *PKS II*, and *NRPS* encoding genes, respectively, in the PCR reactions (Table 5). *PKS-II* (25.9%) and *NRPS* (24.1%) genes exhibited a high positive rate in the members of Streptomyces, while that of *PKS-I* genes was higher in rare actinobacteria (5.6%).

**TABLE 5:** Distribution of antibiotics synthesis genes in 54 antagonistic endophytic actinomycetes.

Taxa group	Number of tested strains	Number of positive strains (%)	Positive results of the PCR test (%)		
			
			PKS I	PKS II	NRPS
Rare actinomycetes	21	8 (14.8)	3 (5.6)	7 (13.0)	2 (3.7)
*Sterptomyces*	33	19 (35.2)	1 (1.6)	14 (25.9)	13 (24.1)
Total	54	27 (50.0)	4 (7.4)	21 (38.9)	15 (27.8)

## Discussion

Endophytic actinobacteria associated with medicinal plants as a biocontrol agent are considered to be beneficial for plant diseases management, and they spend their whole life cycle within plant tissues without causing any detectable infectious or signs of disease symptoms and may also produce the same metabolites and within the living tissues of host plant (Qin et al., 2009; Verma et al., 2009; Passari et al., 2015; Dos Santos et al., 2016). Therefore, these findings encouraged us to investigate for the first time the Chinese traditional medicinal plants *T. roseus* S for understanding the biodiversity and species distribution of the endophytic actinobacteria with respect to different ecological environments and their biosynthetic potential as antimicrobial agents.

The pretreatment of plant tissues is an advantageous method to promote and release rare endophytic actinobacteria from the inner parts of plant tissues. It is an essential and useful step for the isolation of endophytic actinobacteria that is depending on many factors, including host species, geographic and habitat distribution, pretreatment of plants, and selective media (Golinska et al., 2015). In this study, we isolated 126 endophytic actinobacteria from three types of pretreated plant tissues. Among different pretreatments, the number of endophytic actinobacteria with freezing treatment of plant tissues was more than the other two pretreatments. This result indicated that freezing treatment of plant tissues were more useful for the isolation of endophytic actinobacteria and helpful to release rare genera, such as strains T4LB045 and T3RB037, which belong to *Alloactinosynnema* and *Dietzia*. Freezing treatment of plant tissues could inhibit the majority of gram-negative species and the resistibility to low temperatures of spore-forming actinobacteria stronger than the non-spore-forming bacteria (Qin et al., 2011). The current findings indicated that the humic acid-vitamin (HV) medium and sodium propionate agar medium, which supplied by medicinal plant extracts of *T. roseus* S, was the most effective medium for isolating the endophytic actinobacteria due to the fact that the amino acids are the primary nitrogen sources in many plants. This result was in accordance with the previous study of Qin et al. (2009). This also could be due to some physiological characteristics of some actinobacteria in plant tissues being different (Kurtboke, 2003). Moreover, it is worth mentioning the combination of different pretreatment methods, especially freezing pretreatment and media influencing the isolation effectiveness of endophytic actinobacteria.

A total of 126 isolates belonging to 2 classes, 8 orders, 14 families, and 24 genera were isolated from medicinal plants *T. roseus* S from Ili and Tacheng sites in Xinjiang. The results showed a great diversity in populations of endophytic actinobacteria. The diversity analysis showed that the evaluated parameters from each location sample were different; the diversity from Tacheng was more abundant than the Ili site. For example, Venny of endophytic actinobacteria (Figure 3) showed that 5.56% rare genera were only from Tacheng, while 4.76% rare genera were only from the Ili site (Figure 3). Besides, the *Streptomyces* genus was the most abundant genus isolated from two samples (45 out of 126). These data corroborated the results obtained by Qiu et al. (2015), who isolated 119 endophytic actinobacteria 66 of them being affiliated to genus Streptomyces based on 16S rRNA gene sequence analysis. In accordance with these results, previous study has shown that 192, 146, and 159 endophytic actinobacteria were isolated from mangrove plants sampled in the three different natural protection areas Macao, Hainan, and Guangdong, respectively, and only 12 genera were isolated from Macao site (Xue et al., 2013; Li et al., 2016, 2017). In addition, in our previous investigation for isolation of endophytic bacteria associated with medicinal plants in the arid land of Xinjing, Chinam obtained similar observations regarding to geographical environment (Li et al., 2018). However, these results suggested that the select of different geographical environmental habitat to the same host plant to explore the diversity of endophytic actinobacteria resources will helps us to understand more characteristics of the symbiotic relation of host plants with respect to geographical environment of sampled area such as the type of soil and nutrient content (Qin et al., 2011; Govindasamy et al., 2014).

In the current study, *Streptomyces*. sp. was the most dominant in the root tissues, whereas fewer species were isolated from leaves and stem. This finding was in agreement with previous studies of Verma et al. (2009), Kaewkla and Franco (2013), and Kemung et al. (2018); they stated that plant roots provided a suitable habitat for endophytic actinobacteria and genera *Streptomyces* are majority in roots. This may be associated with the fact that the rhizospheric actinobacteria can move along the internal plant tissues and can colonize the roots (Golinska et al., 2015).

The antagonistic activity of the endophytic actinobacteria community associated with medicinal plant *T. roseus* S were screening *in vitro* condition by using a dual culture technique against a diverse range of phytopathogens. The results showed, substantial antagonistic activities against five pathogens, including *Fulvia fulva* (Cooke)Cif., *Alternaria solani*, *Valsa malicola*, *Valsa mali*, and *Salmonella enteritidis*. In contrast, all tested strains showed moderate and weak activities against *Fusarium oxysporum* f.sp. and *Fusarium oxysporum*. The antagonism of endophytic actinobacteria observed in this study was in accordance with previous reports; this includes, for example, the activity of actinobacteria isolated from tomato plants toward a diverse range of phytopathogens, such as *Alternaria solani*, *Fusarium oxyporum* f sp. lycopersici, and *Rhizoctonia* sp. (de Oliveira et al., 2010; Li et al., 2016). In this study, the majority of the 54 antagonistic strains belonged to *Streptomyces.*sp (de Oliveira et al., 2010; Kim et al., 2012; Mingma et al., 2014). Strain T3SB005 belonging to *Streptomyces albidoflavus* showed antagonistic activity against three human pathogens, and it strongly inhibited the growth of *Salmonella enteritidis*. However, Zhang et al. (2015) found that the secondary metabolites of strain *Nocardiopsis dassonvillei* subsp. *dassonvillei* showed potent inhibitory activity against *Staphylococcus aureus*. In addition, strain *Streptomyces* sp. T4SB030 and T4SB028 were able to control many bacterial and fungal pathogens. The inhibition ratio of T4SB028 against *Alternaria solani*, *Valsa malicola*, and *Valsa mali* was 67.06, 64.20, and 70.55%, respectively. This had accordance with the previous study (le Roes-Hill and Meyers, 2009; Fan et al., 2016; Li et al., 2016; Law et al., 2017). This result demonstrated the potential of strains *Streptomyces* sp. T4SB030 and T4SB028 isolated from *T. roseus* S. in the biocontrol of *Alternaria solani*, *Valsa malicola*, and *Valsa mali*. According to our work, the strain T4SB030 and T4SB028 were related to *S. armeniacus*, and *S. polyantibioticus* could be used as putative broad-spectrum biocontrol agents in future studies. Moreover, T3RB033 and T4LB045 might be a new putative antagonistic novel species isolated from *T. roseus*.

Interestingly, strain T4SB004 and T3RB001 with the same taxonomy based on the 16S RNA analysis and isolated from two different geographic and environmental sites exhibited different antagonistic activity. For instance, strain T4SB004 showed antagonistic activity toward the growth of *Valsa malicola*, *Valsa mali*, and *Salmonella enteritidis*, while strain T3RB001 inhibited the growth of *Fusarium oxysporum* f.sp., *Alternaria solani*, and *Fusarium oxysporum*. This indicated that active endophytic strains showed specific selectivity to some pathogens as well as further confirm the fact that the antimicrobial properties of endophytes had a relationship with the geographical site of host plants. In the present study, 30.95%, 23.00% of the tested endophytic actinobacteria were capable of producing siderophores and chitinase, respectively. It is well known that endophytic actinobacteria inhibit the growth of the phytopathogen by competing for iron in the environment (Singh et al., 2017). Besides, chitin is the crucial component of the fungal cell wall. Chitinase, as a fungal cell-wall-degrading enzyme produced by endophytic microorganisms, plays an active role in antagonism (Kalai-Grami et al., 2014; Egamberdieva et al., 2017a). Additionally, the endophytic actinomycete isolates from *Achillea fragrantissima* showed varying degrees of antifungal activity against fungal pathogens and produced chitinases or siderophores, according to El-Shatoury et al. (2009).

In order to understand the biosynthetic potential of the isolates, the detection of genes encoding polyketide synthases and non-ribosomal peptide synthetases, responsible for the synthesis of most biologically active polyketide and peptide compounds, has been broadly used for assessing biosynthetic potential of culturable and non-culturable microorganisms. In this study, at least one antibiotic biosynthetic gene was detected in 27 (representing 50% of tested strains) antagonistic strains. The antimicrobial genes screening results indicated that the presence of *PKS* and *NRPS* encoding genes were widespread in the tested strains. The higher proportions of *PKS II* (38.9 %) and *NRPS* (27.8 %) as well as lower detection rates of *PKS I* (7.4 %) these results were in agreement with the previous study (Lee et al., 2014; Qiu et al., 2015). The absence of amplification of *PKS I*, *PKS II*, and *NRPS* genes in some of the tested strains may be due to the absence of these genes, and the amplified primers were not suitable for screening the biosynthetic genes of some tested strains (Ayuso-Sacido and Genilloud, 2005). Moreover, we obtained the *PKS* and *NRPS* existent strains with other *PKS I*, *PKS II*, and *NRPS* genes primers (Wu et al., 2009). *PKS-II* (25.9%) and *NRPS* (24.1%) genes exhibited high positive rates in the members of *Streptomyces*, and this result was in opposition to the study of Qiu et al. (2015); they argued that the NRPS genes displayed wide distribution in the members of both *Streptomyces* and rare actinobacteria. Among tested endophytes, strains T3L1C, T3RB033, T3SB005, T3SB037, T4LB045, T4LB049, and T4RB005 displayed remarkable antibacterial activity in the bioactive assay, while *PKS* and *NRPS* genes were negative. According Finking and Marahiel (2004), the NRPS genes could also influences quorum-sensing of bacteria, though not in the biosynthesis of bioactive secondary metabolites.

This result suggested that the notion of that there is no direct correlation between the occurrence of biosynthetic genes and the production of antibacterial activities.

## Conclusion

In summary, the results from our pilot study revealed that medicinal plant *T. roseus* Schipcz. provided a rich source of endophytic actinobacteria that exhibited a broad-spectrum antimicrobial agent in *in vitro* conditions. Besides, the diversity of endophytes differed between the plant tissues and geographic locations. These results further confirmed the notion that the pretreatment of plant materials, using selective media and different geographic locations, is an essential step for pure culture isolation. This study showed that bacterial isolates, such as *S. armeniacus* and *S. polyantibioticus*, with antifungal activity could be used as potential biocontrol agents against different kinds of plant pathogens. Furthermore, systematic investigations are needed to explore the chemical diversity of bioactive metabolites produced by these endophytes, which can offer an opportunity to discover novel natural products for the treatment of human diseases as well.

## Data Availability Statement

The 16S rRNA gene sequences determined in this study were deposited in GenBank under accession numbers MN686679–MN686702(24), MN687832–MN687853(22), MN688237–MN688255(19), MN688672–MN688674(3), MN688677(1), MN688679–MN688680(2), MN686608–MN686629(22), MN686648–686678(31), and MN688648–688649(2). Reference sequences used are noted in the phylogenetic trees.

## Author Contributions

LL and W-JL designed the experiments. ZM, GA, and JM conducted the experiments. DE, YL, and OAAM analyzed the data and reviewed the manuscript. ZM, JM, and LL wrote the manuscript. All the authors read and approved the manuscript.

## Conflict of Interest

The authors declare that the research was conducted in the absence of any commercial or financial relationships that could be construed as a potential conflict of interest.

## Footnotes

1 http://www.eztaxon.org
